# Chromatinopathies: insight in clinical aspects and underlying epigenetic changes

**DOI:** 10.1007/s13353-023-00824-1

**Published:** 2024-01-05

**Authors:** Ewelina Bukowska-Olech, Aleksandra Majchrzak-Celińska, Marta Przyborska, Aleksander Jamsheer

**Affiliations:** 1 Department of Medical Genetics, Poznan University of Medical Sciences, Poznan, Poland; 2 Department of Pharmaceutical Biochemistry, Poznan University of Medical Sciences, Poznan, Poland; 3 Centers for Medical Genetics GENESIS, Poznan, Poland

**Keywords:** Chromatin, Episignatures, Epigenes, Methylome, Cardiac defects, Malignancy

## Abstract

Chromatinopathies (CPs), a group of rare inborn defects characterized by chromatin state imbalance, have evolved from initially resembling Cornelia de Lange syndrome to encompass a wide array of genetic diseases with diverse clinical presentations. The CPs classification now includes human developmental disorders caused by germline mutations in epigenes, genes that regulate the epigenome. Recent advances in next-generation sequencing have enabled the association of 154 epigenes with CPs, revealing distinctive DNA methylation patterns known as episignatures.

It has been shown that episignatures are unique for a particular CP or share similarities among specific CP subgroup. Consequently, these episignatures have emerged as promising biomarkers for diagnosing and treating CPs, differentiating subtypes, evaluating variants of unknown significance, and facilitating targeted therapies tailored to the underlying epigenetic dysregulation.

The following review was conducted to collect, summarize, and analyze data regarding CPs in such aspects as clinical evaluation encompassing long-term patient care, underlying epigenetic changes, and innovative molecular and bioinformatic methodologies that have been devised for the assessment of CPs. We have also shed light on promising novel treatment options that have surfaced in recent research and presented a synthesis of ongoing clinical trials, contributing to the current understanding of the dynamic and evolving nature of CPs investigation.

**Supplementary Information:**

The online version contains supplementary material available at 10.1007/s13353-023-00824-1.

## Introduction

Chromatinopathies (CPs) are rare inborn defects resulting from chromatin state imbalance (Bjornsson 2015; Fahrner and Bjornsson 2019). The term was coined initially to describe conditions that resemble Cornelia de Lange syndrome (MIM:122470, 300590, 610759, 614701, 300882), however, were caused by genes different from those in the cohesin complex, such as *NIPBL*, *SMCA1*, *SMC3*, *RAD21*, and *HDAC8* (Parenti and Kaiser 2021; Avagliano et al. 2020; Bjornsson 2015). Next, the CPs definition was extended to a wide range of genetic diseases with diverse clinical presentations, including facial dysmorphism, neurodevelopmental and cardiac abnormalities, immunodeficiency, and cancer predisposition. Finally, Nava and Arboleda have proposed that CPs should refer to each human developmental disorder resulting from germline mutations in genes that regulate epigenome, i.e., epigenes (Nava and Arboleda 2023).

Epigenes encode proteins that maintain the epigenome. Depending on their function, these proteins were classified into four groups—(1) chromatin modifiers, (2) chromatin remodelers, (3) proteins modulating DNA/RNA chemical modifications, and (4) accessory proteins (Sadakierska-Chudy et al. 2015; Sadakierska-Chudy and Filip 2015; Javaid and Choi 2017). Nava and Arboleda, however, presented the extended classification in which 17 groups of these proteins were listed—(1) histone writers, (2) histone erasers, (3) histone readers, (4) chromatin remodelers, (5) histone chaperones, (6) scaffold proteins, (7) DNA modifiers, (8) RNA modifiers, (9) polycomb group proteins, (10) transcription factors, (11) proteins cofactor for histone writer, (12) proteins cofactor for histone eraser, (13) proteins cofactor for histone reader, (14) proteins cofactor for chromatin remodeler, (15) proteins cofactor for histone chaperone, (16) proteins cofactor for DNA modifier, and (17) proteins cofactor for RNA modifier (Nava and Arboleda 2023). A detailed characterization of these proteins is presented in Table 1.

**Table 1:** Characterization of 17 protein classes encoded by epigenes (Nava and Arboleda 2023)

	Function	Encoding epigenes linked with chromatinopathies
Histone modification writers	(a) Histone acetylases; proteins capable of adding acetyl groups to histones (b) Histone methyltransferases; proteins capable of adding methyl groups to histones	*ARID1B*, *ASH1L*, *ATM*, *ATR*, *BRCA2*, *CHUK*, *CREBBP*, *CUL3*, *CULB4*, *DDB1*, *EHMT1*, *EP300*, *HUWE1*, *KAT5*, *KAT6A*, *KAT6B*, *KAT8*, *KMT2A*, *KMT2C, KMT2D, KMT2E, KMT5B*, *MAP3K7*, *NEK9*, *NSD1*, *NSD2*, *OGT*, *PPP2CA*, *PRMT7*, *RAD51*, *RAG1*, *RNF168*, *RNF2*, *SETD1A*, *SETD1B*, *SETD2*, *SETD5*, *SPOP*, *TAF1*, *TLK2*, *UBE2A*, *VRK1*
Histone modification erasers	(a) Histone deacetylases; proteins capable of removing acetyl groups from histones (b) Histone demethylases; proteins capable of removing methyl groups from histones	*BAP1*, *EYA1*, *HDAC4*, *HDAC8*, *KDM1A*, *KDM3B*, *KDM5B*, *KDM5C*, *KDM6A, KDM6B*, *MYSM1*, *PHF8*, *SRCAP*
Histone modification readers	Proteins capable of identifying histone post-translational modifications	*AIRE, ASXL2*, *GATAD2B*, *CRB2*, *MSH6*, *MSL3*, *PHIP*, *POGZ*, *RAG2*, *SMARCA4*, *SMARCB1*, *SUPT16H*, *ZMYND11*
Chromatin remodelers	Proteins capable of catalyzing a broad range of chromatin changing reactions including sliding of an octamer across the DNA (nucleosome sliding), changing the conformation of nucleosomal DNA and altering the composition of the octamers (histone variant exchange)	*ATRX*, *BPTF*, *CDC6*, *CHD1*, *CHD2*, *CHD3*, *CHD4*, *CHD7*, *CTBP1*, *CTCF*, *DPF2*, *ERCC6*, *HCFC1*, *HELLS*, *MBD5*, *MYSM1*, *PCNA*, *RAI1*, *SMARCAD1*, *SMARCD1*, *SRCAP*
Histone chaperones	Histone-binding proteins that influence chromatin dynamics in an ATP-independent manner	*TAF6*
Scaffold proteins	Proteins capable of bringing together two or more proteins in a relatively stable configuration	*ASXL3*, *ELP1*, *EXOSC2*
DNA modifiers	Proteins capable of regulating DNA structure	*AICDA*, *DNMT1*, *DNMT3A*, *DNMT3B*, *TET3*, *USP7*
RNA modifiers	Proteins capable of regulating RNA structure	*EXOSC2*
Polycomb group proteins	Proteins capable of genes silencing	*ASXL1*, *ASXL3*, *BAP1*, *BCOR*, *EED*, *EZH2*, *PCGF2*, *SUZ12*
Transcription factors	Proteins capable of gene expression regulations	*AIRE*, *BRCA1*, *CTCF*, *FOXP1*, *FOXP2*, *FOXP3*, *MECP2*, *SIN3A*, *SMARCA2*, *SMARCA4*, *SUZ12*, *TAF2*, *TP53*, *VDR*, *YY1*
Cofactors for histone writers	Histone writers accessory proteins	*BRCA1*, *CDC73*, *KANSL1*, *LAS1L*, *MECP2*, *MEN1*, *PRKAG2*, *RPS6KA3*, *TP53*, *TRRAP*, *WAC*
Cofactors for histone erasers	Histone erasers accessory proteins	*ATN1*, *BCORL1*, *SIN3A*, *SNAI2*, NIPBL, *PHF21A*, *RLIM*, *ZNF111*
Cofactors for histone modification readers	Histone readers accessory proteins	n.a.
Cofactors for chromatin modification remodelers	Chromatin modification remodelers accessory proteins	*ACTB*, *ACTL6B*, *ADNP*, *ARID1A*, *ARID2*, *SATB1*, *SMARCC2*, *SMARCE1*, *VDR*, *YY1*
Cofactors for histone chaperones	Histone chaperons accessory proteins	n.a.
Cofactors for DNA modifiers	DNA modifiers accessory proteins	*USP7*
Cofactors for RNA modifiers	RNA modifiers accessory proteins	n.a.

*n.a.*, not applicable

Next-generation-based methods have enabled researchers to associate 154 out of approximately 300 epigenes with CPs (Squeo et al. 2020; Boukas et al. 2019; Kerkhof et al. 2022; Bukowska-Olech et al. 2022; Zollino et al. 2017; Nava and Arboleda 2023). Recent studies have revealed that variants in epigenes lead to distinctive and disorder-specific DNA methylation patterns, known as episignatures (Levy et al. 2021; Beck et al. 2020; Sadikovic et al. 2020). Episignatures are promising biomarkers for the diagnosis and treatment of CPs as they can differentiate various CP subtypes linked to specific gene variants and may enable the development of targeted therapies tailored to the disease-causing epigenetic dysregulation (Nava and Arboleda 2023; Vos et al. 2023; Kerkhof et al. 2022).

## Clinical manifestation and common phenotypic features

The prime example of CPs is Cornelia de Lange syndrome (MIM: # 122470, #300590, #610759, #614701, and #300882), a multisystemic disorder that may encompass variable clinical features such as growth retardation, craniofacial abnormalities, intellectual disability, hirsutism, genitourinary defects, limb defects, gastroesophageal reflux, or heart defects (Piché et al. 2019; Kline et al. 2007; Sarogni et al. 2020). As the disease phenotype may be heterogeneous, international recommendations for improving Cornelia de Lange syndrome and its management were proposed. Both cardinal (synophrys and/ or thick eyebrows, short nose, concave nasal ridge and/or upturned nasal tip, long and/ or smooth philtrum, thin upper lip vermilion, downturned corners of the mouth, hand oligodactyly and/ or adactyly, and congenital diaphragmatic hernia) and suggestive (global developmental delay and/ or intellectual disability, prenatal growth retardation, postnatal growth retardation, microcephaly (prenatally and/ or postnatally), small hands and/ or feet, short fifth finger, and hirsutism) features were listed. If present, each cardinal feature is scored with 2 points, whereas a score of 1 point is assigned to each suggestive feature. A total score of ≥ 11 points (with at least 3 cardinal) indicates a classic Cornelia de Lange syndrome; 9 or 10 points (at least 2 cardinal) is suggestive of a non-classic Cornelia de Lange syndrome; 4 to 8 points (at least 1 cardinal) requires molecular testing, whereas a score of fewer than 4 points is insufficient to indicate further exams (Kline et al. 2007).

Currently, 179 syndromes are classified as CPs, as shown in Table 2. Clinical manifestations and common phenotypic features of CPs can vary depending on the underlying genetic mutation and its impact on chromatin regulation. However, some general clinical characteristics are observed across different CP subtypes. These include neurodevelopmental delay and intellectual disability, seizures, facial dysmorphism, growth and feeding difficulties, and abnormalities of heart or skeleton (Parenti and Kaiser 2021; Fazio et al. 2019; Björkman et al. 2018; Boot et al. 2018a; Karagianni et al. 2016; Litwin and Wysocki 2018; Aukema et al. 2023; Ciptasari and van Bokhoven 2020). CP syndromes often require long-term monitoring as the phenotype changes over time. Furthermore, individuals with CPs may also experience additional health challenges, such as immunodeficiency and an increased predisposition to cancer (Boot et al. 2018a; Laufer et al. 2019; Vos et al. 2023; Fazio et al. 2019; Boniel et al. 2021a; Björkman et al. 2018; Szczawinska-Poplonyk et al. 2022).

**Table 2:** The list of chromatinopathies syndromes and linked epigenes (based on OMIM database)

	Chromatinopathy syndrome	OMIM	Epigene	Inheritance
1	Alazami-Yuan syndrome	617126	*TAF6*	AR
2	Alpha-thalassemia/impaired intellectual development syndrome, X-linked	301040	*ATRX*	XLD
3	Arboleda-Tham syndrome	616268	*KAT6A*	AD
4	Ataxia-teleangiectasia	208900	*ATM*	AR
5	Ataxia-teleangiectasia-like disorder 2	615919	*PCNA*	AR
6	Autoimmune lymphoproliferative syndrome type III	615559	*PRKCD*	AR
7	Autoimmune polyendocrine syndrome, type 1, with or without reversible metaphyseal dysplasia	240300	*AIRE*	AD/AR
8	Bainbridge-Ropers syndrome	615485	*ASXL3*	AD
9	Baraitser-Winter syndrome 1	243310	*ACTB*	AD
10	Bartsocas-Papas syndrome 2	619339	*CHUK*	AR
11	Basan syndrome	612761	*SMARCAD1*	AD
12	Basilicata-Akhtar syndrome	301032	*MSL3*	XLD
13	Beck-Fahrner syndrome	618798	*TET3*	AD/AR
14	Blepharophimosis-impaired intellectual development syndrome	619293	*SMARCA2*	AD
15	Bohring-Opitz syndrome	605039	*ASXL1*	AD
16	Bone marrow failure syndrome 4	618116	*MYSM1*	AR
17	Bone marrow failure syndrome 5	618165	*TP53*	AD
18	Branchiootic syndrome 1	602588	*EYA1*	AD
19	Branchiootorenal syndrome 1	113650	*EYA1*	AD
20	Cardiospondylocarpofacial syndrome	157800	*MAP3K7*	AD
21	Cerebellar ataxia, deafness, and narcolepsy, autosomal dominant	604121	*DNMT1*	AD
22	Cerebrooculofacioskeletal syndrome 1	214150	*ERCC6*	AR
23	CHARGE syndrome	214800	*CHD7*	AD
24	Chromosome 2q37 deletion syndrome	600430	*HDAC4*	AD
25	Chung-Jansen syndrome	617991	*PHIP*	AD
26	Cleft palate, psychomotor retardation, and distinctive facial features	616728	*KDM1A*	AD
27	Cockayne syndrome B	133540	*ERCC6*	AR
28	Coffin-Lowry syndrome	303600	*RPS6KA3*	XLD
29	Coffin-Siris syndrome 1	135900	*ARID1B*	AD
30	Coffin-Siris syndrome 2	614607	*ARID1A*	AD
31	Coffin-Siris syndrome 3	614608	*SMARCB1*	AD
32	Coffin-Siris syndrome 4	614609	*SMARCA4*	AD
33	Coffin-Siris syndrome 5	616938	*SMARCE1*	AD
34	Coffin-Siris syndrome 6	617808	*ARID2*	AD
35	Coffin-Siris syndrome 7	618027	*DPF2*	AD
36	Coffin-Siris syndrome 8	618362	*SMARCC2*	AD
37	Coffin-Siris syndrome 11	618779	*SMARCD1*	AD
38	Cohen-Gibson syndrome	617561	*EED*	AD
39	Cornelia de Lange syndrome 1	122470	*NIPBL*	AD
40	Cornelia de Lange syndrome 5	300882	*HDAC8*	XLD
41	Cutaneous teleangiectasia and cancer syndrome, familial	614564	*ATR*	AD
42	de Sanctis-Cacchione syndrome	278800	*ERCC6*	AR
43	Dentatorubral-pallidoluysian atrophy	125370	*ATN1*	AD
44	Desanto-Shinawi syndrome	616708	*WAC*	AD
45	Developmental and epileptic encephalopathy 76	618468	*ACTL6B*	AR
46	Developmental and epileptic encephalopathy 94	615369	*CHD2*	AD
47	Developmental delay with dysmorphic facies and dental anomalies	619228	*SATB1*	AD
48	Developmental delay with or without dysmorphic facies and autism	618454	*TRRAP*	AD
49	Developmental delay, hypotania, musculoskeletal defects, and behavioral abnormalities	619595	*SRCAP*	AD
50	Diets-Jongmans syndrome	618846	*KDM3B*	AD
51	Dystonia-Parkinsonism, X-linked	314250	*TAF1*	XLR
52	Epilepsy, early-onset, 2, with or without developmental delay	618832	*SETD1A*	AD
53	Fanconi anemia, complementation group D1	605724	*BRCA2*	AR
54	Fanconi anemia, complementation group R	617244	*RAD51*	AD
55	Fanconi anemia, complementation group S	617883	*BRCA1*	AR
56	Fetal encasement syndrome	613630	*CHUK*	AR
57	Floating-Harbor syndrome	136140	*SRCAP*	AD
58	Focal segmental glomerulosclerosis 9	616220	*CRB2*	AR
59	Gabriele-de Wries syndrome	617557	*YY1*	AD
60	Gand syndrome	615074	*GATAD2B*	AD
61	Genitopatellar syndrome	606170	*KAT6B*	AD
62	Glass syndrome	612313	*SATB2*	AD
63	Hao-Fountain syndrome	616863	*USP7*	AD
64	Helsmoortel-van der AA syndrome	615873	*ADNP*	AD
65	Heyn-Sproul-Jackson syndrome	618724	*DNMT3A*	AD
66	Huriez syndrome	181600	*SMARCAD1*	AD
67	Hyperparathyroidism-jaw tumor syndrome	145001	*CDC73*	AD
68	Hypogonadotropic hypogonadism 5 with or without anosmia	612370	*CHD7*	AD
69	Hypotonia, ataxia, developmental delay, and tooth enamel defect syndrome	617915	*CTBP1*	AD
70	Imagawa-Matsumoto syndrome	618786	*SUZ12*	AD
71	Immunodeficiency-centromeric instability-facial anomalies syndrome 1	242860	*DNMT3B*	AR
72	Immunodeficiency-centromeric instability-facial anomalies syndrome 4	616911	*HELLS*	AR
73	Immunodeficiency with hiper-IgM, type 2	605258	*AICDA*	AR
74	Immunodeficiency, X-linked, with hiper-IgM	308230	*TNFSF5*	XLR
75	Immunodysregulation, polyendocrinopathy, and enteropathy, X-linked syndrome	304790	*FOXP3*	XLR
76	Intellectual developmental disorder with behavioral abnormalities and craniofacial dysmorphism with or without seizures	618725	*PHF21A*	AD
77	Intellectual developmental disorder with dysmorphic facies and ptosis	617333	*BRPF1*	AD
78	Intellectual developmental disorder with language impairment and with or without autistic features	613670	*FOXP1*	AD
79	Intellectual developmental disorder with severe speech and ambulation defects	618470	*ACTL6B*	AD
80	Intellectual developmental disorder X-linked, syndromic, Lubs type	300260	*MECP2*	XLR
81	Intellectual developmental disorder, autosomal dominant 1	156200	*MBD5*	AD
82	Intellectual developmental disorder, autosomal dominant 21	615502	*CTCF*	AD
83	Intellectual developmental disorder, autosomal dominant 23	615761	*SETD5*	AD
84	Intellectual developmental disorder, autosomal dominant 30	608668	*ZMYND11*	AD
85	Intellectual developmental disorder, autosomal recessive 40	615599	*TAF2*	AR
86	Intellectual developmental disorder, autosomal dominant 41	616944	*TBL1XR1*	AD
87	Intellectual developmental disorder, autosomal dominant 51	617788	*KMT5B*	AD
88	Intellectual developmental disorder, autosomal dominant 52	617796	*ASH1L*	AD
89	Intellectual developmental disorder, autosomal dominant 57	618050	*TLK2*	AD
90	Intellectual developmental disorder, autosomal dominant 58	618106	*SET*	AD
91	Intellectual developmental disorder, autosomal dominant 65	619320	*KDM4B*	AD
92	Intellectual developmental disorder, autosomal recessive 65	618109	*KDM5B*	AR
93	Intellectual developmental disorder with seizures and language delay	619000	*SETD1B*	AD
94	Intellectual developmental disorder, X-linked 33	300966	*TAF1*	XLR
95	Intellectual developmental disorder, X-linked 41	300849	*GDI1*	XLD
96	Intellectual developmental disorder, X-linked, syndromic 13	300055	*MECP2*	XLR
97	Intellectual developmental disorder, X-linked, syndromic, Cabezas type	300354	*CUL4B*	AD
98	Intellectual developmental disorder, X-linked, syndromic, Claes-Jensen type	300534	*KDM5C*	XLR
99	Intellectual developmental disorder, X-linked, syndromic, Nascimento type	300860	*UBE2A*	XLR
100	Intellectual developmental disorder, X-linked, syndromic, siderius type	300263	*PHF8*	XLR
101	Intellectual developmental disorder, X-linked, syndromic, Turner type	309590	*HUWE1*	XL
102	Intellectual developmental disorder, X-linked, syndromic, Wilson-Turner type	309585	*LAS1L*	XLR
103	Intellectual disability-hypotonic facies syndrome, X-linked	309580	*ATRX*	XLR
104	Kabuki syndrome 1	147920	*KMT2D*	AD
105	Kabuki syndrome 2	300867	*KDM6A*	XLD
106	Kleefstra syndrome 1	610253	*EHMT1*	AD
107	Kleefstra syndrome 2	617768	*KMT2C*	AD
108	Kohlschutter-Tonz syndrome-like	226750	*ROGDI*	AR
109	Koolen-de Vries syndrome	610443	*KANSL1*	AD
110	Lethal congenital contracture syndrome 10	617022	*NEK9*	AD
111	Li-Fraumeni syndrome	151623	*TP53*	AD
112	Li-Ghorbani-Weisz-Hubshman syndrome	618974	*KAT8*	AD
113	Luo-Schoch-Yamamoto syndrome	619460	*RNF2*	AD
114	Luscan-Lumisch syndrome	616831	*SETD2*	AD
115	Meier-Gorlin syndrome 5	613805	*CDC6*	AR
116	Menke-Hennekam syndrome 1	618332	*CREBBP*	AD
117	Menke-Hennekam syndrome 2	618333	*EP300*	AD
118	Microphthalmia syndromic 2	300166	*BCOR*	XLD
119	Mismatch repair cancer syndrome 3	619097	*MSH6*	AR
120	Mismatch repair cancer syndrome 4	619101	*PMS2*	AR
121	Multiple endocrine neoplasia type I	131100	*MEN1*	AD
	Nabais Sa-de Vries syndrome type 1	618828	*SPOP*	AD
122	Nabais Sa-de Vries syndrome type 2	618829	*SPOP*	AD
123	Neurodevelopmental disorder with dysmorphic facies and distal limb anomalies	617755	*BPTF*	AD
124	Neurodevelopmental disorder and language delay with or without structural brain abnormalities	618354	*PPP2CA*	AD
125	Neurodevelopmental disorder with coarse facies and mild distal skeletal abnormalities	618505	*KDM6B*	AD
126	Neurodevelopmental disorder with dysmorphic facies and thin corpus callosum	619480	*SUPT16H*	AD
127	Neurodevelopmental disorder with dysmorphic facies, sleep disturbance, and brain abnormalities	619103	*KAT5*	AD
128	Neurodevelopmental disorder with hypotonia, impaired language, and dysmorphic features	616579	*CHAMP1*	AD
129	Neurodevelopmental disorder with or without autism and seizures	619239	*CUL3*	AD
130	Neurodevelopmental disorder with speech impairment and dysmorphic facies	619056	*SETD1A*	AD
131	Neuropathy, hereditary sensory, and autonomic type III	223900	*ELP1*	AR
132	Neuropathy, hereditary sensory, type IE	614116	*DNMT1*	AD
	Nicolaides-Baraitser syndrome	601358	*SMARCA2*	AD
133	Nijmegen breakage syndrome	251260	*NBN*	AR
134	O'Donnell-Luria-Rodan syndrome	618512	*KMT2E*	AD
135	Ohdo syndrome, SBBYS variant	603736	*KAT6B*	AD
136	Okur-Chung neurodevelopmental syndrome	617062	*CSNK2A1*	AD
137	Omenn syndrome	603554	*RAG1, RAG2*	AR
138	Pierpont syndrome	602342	*TBL1XR1*	AD
139	Pilarowski-Bjornsson syndrome	617682	*CHD1*	AD
140	Pontocerebellar hypoplasia type 1A	607596	*VRK1*	AR
141	Rauch-Steindl syndrome	619695	*NSD2*	AD
142	Rett syndrome	312750	*MECP2*	XLD
143	Reynolds syndrome	613471	*LBR*	AD
144	Rhabdoid tumor predisposition syndrome 1	609322	*SMARCB1*	AD
145	Rhabdoid tumor predisposition syndrome 2	613325	*SMARCA4*	AD
146	Rickets, vitamin D resistant, type IIA	277440	*VDR*	AR
147	Riddle syndrome	611943	*RNF168*	AR
148	Rubinstein-Taybi syndrome 1	180849	*CREBBP*	AD
149	Rubinstein-Taybi syndrome 2	613684	*EP300*	AD
150	Seckel syndrome 1	210600	*ATR*	AR
152	Shashi-Pena syndrome	617190	*ASXL2*	AD
153	Short stature, brachydactyly, impaired intellectual development and seizures	617157	*PRMT7*	AR
154	Short stature, hearing loss, retinitis pigmentosa, and distinctive facies	617763	*EXOSC2*	AR
155	Shukla-Vernon syndrome	301029	*BCORL1*	XLR
156	Sifrim-Hitz-Weiss syndrome	617159	*CHD4*	AD
157	Smith-Magenis syndrome	182290	*RAI1*	AD
158	Snijders Blok-Campeu syndrome	618205	*CHD3*	AD
159	Sotos syndrome	117550	*NSD1*	AD
160	Speech and language disorder 1	602081	*FOXP2*	AD
161	Tatton-Brown-Rahman syndrome	615879	*DNMT3A*	AD
162	Tonne-Kalscheuer syndrome	300978	*RLIM*	XL
163	Tumor predisposition syndrome	614327	*BAP1*	AD
164	Turnpenny-Fry syndrome	618371	*PCGF2*	AD
165	Uncombable hair syndrome 1	191480	*PADI3*	AR
166	UV-sensitive syndrome 1	600630	*ERCC6*	AR
167	Ventriculomegaly with cystic kidney disease	219730	*CRB2*	AR
168	Waardenburg syndrome type 2A	193510	*MITF*	AD
169	Weaver syndrome	277590	*EZH2*	AD
170	White-Kernohan syndrome	619426	*DDB1*	AD
171	White-Sutton syndrome	616364	*POGZ*	AD
172	Wiedemann-Steiner syndrome	605130	*KMT2A*	AD
173	Witteveen-Kolk syndrome	613406	*SIN3A*	AD
174	Wolff-Parkinson-White syndrome	194200	*PRKAG2*	AD
175	Wolf-Hirschorn syndrome	194190	*CTBP1*	AD
176	X-linked intellectual developmental disorder 19	300844	*RPS6KA3*	XLD
177	X-linked intellectual developmental disorder 93	300659	*BRWD3*	XLR
178	X-linked intellectual developmental disorder 97	300803	*ZNF711*	XL
179	X-linked intellectual developmental disorder 106	300997	*OGT*	XLR

*AD*, autosomal dominant; *AR*, autosomal recessive; *XLD*, X-linked dominant; *XLR*, *X*-linked recessive

Early detection of heart defects, malignancy, immunodeficiency, and other potentially treatable features is paramount to ensure that individuals receive the requisite medical attention and interventions needed to alleviate the severity of these conditions and enhance their overall health and well-being. Therefore, clinicians must be aware of these conditions’ risk development and carefully evaluate them within CP patients clinical follow-up (Table 3).

**Table 3:** List of conditions noted in chromatinopathy syndromes requiring early identification and proper management (based on OMIM database)

	Chromatinopathy syndrome
Malignancy	Ataxia-telangiectasia Ataxia-telangiectasia-like disorder 2 Autoimmune polyendocrine syndrome, type 1, with or without reversible metaphyseal dysplasia Cutaneous telangiectasia and cancer syndrome, familial Fanconi anemia, complementation group D1 Fanconi anemia, complementation group S Huriez syndrome Hyperparathyroidism-jaw tumor syndrome Kabuki syndrome 1 and 2 Li-Fraumeni syndrome mismatch repair cancer syndrome 3 and 4 Multiple endocrine neoplasia type I Nijmegen breakage syndrome Rhabdoid tumor predisposition syndrome 1 and 2 Tatton-Brown-Rahman syndrome
Immunodeficiency	Ataxia-telangiectasia Autoimmune lymphoproliferative syndrome type III Autoimmune polyendocrine syndrome, type 1, with or without reversible metaphyseal dysplasia Cutaneous telangiectasia and cancer syndrome, familial Fanconi anemia, complementation group D1 Helsmoortel-van der AA syndrome Heyn-Sproul-Jackson syndrome Immunodeficiency-centromeric instability-facial anomalies syndrome 1 and 4 Immunodeficiency with hiper-IgM, type 2 Immunodeficiency, X-linked, with hiper-IgM Immunodysregulation, polyendocrinopathy, and enteropathy, X-linked syndrome Kabuki syndrome 1 Menke-Hennekam syndrome 2 Mismatch repair cancer syndrome 3 and 4 Neurodevelopmental disorder with speech impairment and dysmorphic facies Nijmegen breakage syndrome Okur-Chung neurodevelopmental syndrome Omenn syndrome Pilarowski-Bjornsson syndrome Riddle syndrome Rubinstein-Taybi syndrome 1 Turnpenny-Fry syndrome
Heart defects	Alpha-thalassemia/impaired intellectual development syndrome, X-linked Arboleda-Tham syndrome Bohring-Opitz syndrome Cardiospondylocarpofacial syndrome CHARGE syndrome Cockayne syndrome B Coffin-Lowry syndrome Coffin-Siris syndrome 2-5, 7 Cohen-Gibson syndrome Cornelia de Lange syndrome 1 Desanto-Shinawi syndrome Developmental delay with dysmorphic facies and dental anomalies Developmental delay with or without dysmorphic facies and autism Fanconi anemia, complementation group D1 Floating-Harbor syndrome Gand syndrome Genitopatellar syndrome Helsmoortel-van der AA syndrome Intellectual developmental disorder, autosomal dominant 21 Kabuki syndrome 1 and 2 Koolen-de Vries syndrome Microphthalmia syndromic 2 Nabais Sa-de Vries syndrome type 2 Neurodevelopmental disorder and language delay with or without structural brain abnormalities Neurodevelopmental disorder with coarse facies and mild distal skeletal abnormalities Neurodevelopmental disorder with dysmorphic facies and thin corpus callosum Neurodevelopmental disorder with or without autism and seizures Ohdo syndrome, SBBYS variant Rett syndrome Rubinstein-Taybi syndrome 1 Sifrim-Hitz-Weiss syndrome Smith-Magenis syndrome Snijders Blok-Campeu syndrome Sotos syndrome Tatton-Brown-Rahman syndrome Tonne-Kalscheuer syndrome Turnpenny-Fry syndrome

### Congenital heart defects

It has been shown that congenital heart defects noted in CPs may encompass a wide spectrum of abnormalities such as (1) tetralogy of Fallot (Coffin-Siris syndrome, CHARGE syndrome, Sifrim-Hitz-Weiss syndrome, Kleefstra syndrome, and Cornelia de Lange syndrome), (2) atrial septal defect (Coffin-Siris syndrome, Sifirim-Hitz-Weiss syndrome, Kabuki syndrome, Kleefstra syndrome, Wolf-Hirschorn syndrome, Rubinstein-Taybi syndrome, Microphthalmia syndromic 2, Cornelia de Lange syndrome, Intellectual disability-hypotonic facies syndrome X-linked, and Immunodeficiency-centromeric instability-facial anomalies syndrome 1), (3) ventricular septal defect (Coffin-Siris syndrome, CHARGE syndrome, Sifrim-Hitz-Weiss syndrome, Kabuki syndrome, Kleefstra syndrome, Rubinstein-Taybi syndrome, Microphthalmia syndromic 2, Cornelia de Lange syndrome, and Immunodeficiency-centromeric instability-facial anomalies syndrome 1), (4) interrupted aortic arch (CHARGE syndrome), (5) pulmonary stenosis (CHARGE syndrome, Kleefstra syndrome, Cornelia de Lange syndrome, and Intellectual disability-hypotonic facies syndrome X-linked), (6) aortic stenosis (Intellectual disability-hypotonic facies syndrome X-linked), (7) patent ductus arteriosus (Coffin-Siris syndrome, CHARGE syndrome, Sifrim-Hitz-Weiss syndrome, Cornelia de Lange syndrome, and Intellectual disability-hypotonic facies syndrome X-linked), (8) mitral valve anomalies (Microphthalmia syndromic 2), and (9) double-outlet right ventricle (CHARGE syndrome) (Linglart and Bonnet 2022).

Congenital heart defects associated with CPs may be obscured by the underlying disease, leading to under-recognition and inadequate treatment. Additionally, limited knowledge exists regarding conduction heart abnormalities, although they can be life-threatening if untreated. Therefore, it is essential to conduct electrocardiography (EKG) and echocardiography at the time of genetic diagnosis and subsequently as part of the follow-up evaluation (Boniel et al. 2021b; Digilio et al. 2001; Digilio et al. 2017).

### Immunodeficiencies

Epigenes linked to CPs are involved in immunologic processes. Therefore, a comprehensive immunological investigation is imperative, followed by the implementation of specific supportive treatments. Notably, mutations in Kabuki syndrome-related genes, i.e., *KMT2D* and *KDM6A,* lead to impaired B-lymphocyte terminal differentiation. This results in hypogammaglobulinemia and diminished memory B-cell numbers, culminating in common variable immunodeficiency (CVID) (Szczawinska-Poplonyk et al. 2022; Boniel et al. 2021b). In the context of Cornelia de Lange syndrome, genes associated with the condition play a crucial role in maintaining immune system balance. Consequently, patients often contend with chronic ear infections, persistent viral respiratory infections, pneumonia, sinus infections, oral candidiasis, sepsis, and bacterial skin infections (Jyonouchi et al. 2013). Individuals affected by CHARGE syndrome may exhibit a spectrum of immunologic defects. These range from asymptomatic alterations in absolute T-cell counts to severe combined immunodeficiency (SCID) (Mehr et al. 2017). Regarding Kleefstra syndrome, it has been shown that pulmonary infections are more severe in individuals with larger 9q34 deletions than in those with smaller deletions or intragenic *EHMT1* pathogenic variants (Deciphering Developmental Disorders Study 2017). Conversely, Rubinstein-Taybi syndrome typically presents with B-cell defects, T-cell defects, immune dysregulation (autoimmunity and lymphoproliferation), or SCID (Saettini et al. 2020).

### Malignancy

It has been demonstrated that in certain CP syndromes, besides those known to have a higher predisposition to cancer, malignant tumorogenesis can also occur. First, esophagus cancer constitutes the most often occurring cancer type in Cornelia de Lange syndrome patients; however, lymphoblastic leukemia, acute megakaryoblastic leukemia, endometrial carcinoma, gastric cancer, intracranial germinoma, lymphoma, liver hemangioendothelioma, pancreatic neuroendocrine/ endometrial cancer, sacrococcygeal teratoma, suprasellar germinoma, or Wilms tumor have been also reported (Pallotta et al. 2023). Second, several Kabuki syndrome patients with hematological malignancies were reported including acute lymphocytic leukemia, Hodgkin’s lymphoma, or Burkitt lymphoma. Solid tumors, however, encompass giant cell fibroblastoma, or tanycytic spinal ependymoma (Boniel et al. 2021b). Third, Coffin-Siris syndrome has been associated with medulloblastoma, neuroblastoma, schwannomatosis, hepatoblastoma, acute lymphoblastic leukemia, Sertoli-Leydig cell tumor, temporal glioneuronal tumor, papillary thyroid carcinoma, small-cell carcinoma of the ovary, hypercalcemic type, and anaplastic astrocytoma (Borja et al. 2023). Finally, medulloblastoma, neuroblastoma, glioma, neuroma, neurilemmoma, pheochromocytoma, diffuse large-cell B-cell lymphoma, breast cancer, breast adenocarcinoma, hepatoblastoma, parathyroid adenoma, thymoma, thyroid cancer, non-small cell lung carcinoma, acute lymphoblastic leukemia, acute megakaryoblastic leukemia, colon carcinoma, colon cancer, leiomyosarcoma omentum, leiomyosarcoma, rhabdomyosarcoma, granular cell tumor, fibrolipoma, endometrium adenocarcinoma, and serous ovarian carcinoma were noted within Rubinstein-Taybi syndrome. Moreover, patients affected with this syndrome are at increased risk of developing benign meningiomas and pilomatricoma (Boot et al. 2018b).

## Treatment options

Current treatment options for CP patients include supportive care and preventive screening. Typically, neurodevelopmental and neurobehavioral manifestations can be effectively managed, as outlined by clinicians from the Epigenetic and Chromatin Clinic at Johns Hopkins. These interventions encompass occupational therapy, physical therapy, speech and language therapy, behavioral therapy, and the administration of drugs for conditions such as ADHD, anxiety, and epilepsy (Harris et al. 2023). Screening or annual evaluation tests should be applied for syndromes presenting a higher risk of malignancy, cardiac defects, or immunodeficiencies (Table 3), followed by initiation of appropriate therapy, which may include pharmacotherapy, surgical interventions, radiotherapy, and chemotherapy (Boniel et al. 2021a; Björkman et al. 2018; Karagianni et al. 2016; Verrotti et al. 2013; Fazio et al. 2019; Boot et al. 2018a).

Efforts to design targeted treatments for CPs have already commenced. Supplementary Table 1 presents a detailed list of ongoing or completed clinical trials focusing on various CPs or imprinting defects. Therapies targeting epigenetic marks already exist in the case of different forms of cancer, where epigenetics plays a crucial role (Majchrzak-Celińska et al. 2021). However, no targeted therapies have received FDA approval for treating CPs yet (Harris et al. 2023). Nevertheless, promising data from epigenetic-based therapies for imprinting defects were reported. First, epigenetic modification can be achieved by (1) small molecules targeting histone deacetylases (HDAC) such as vorinostat, belinostat, romidepsin, tucidinostat, and panabinostat. Small molecule drug, i.e., topotecan, a topoisomerase inhibitor, has been demonstrated to reactivate the abnormally silenced allele in an Angelman syndrome (AS) mouse model (Huang et al. 2011). EHMT2/ G9a inhibitors reducing H3K9me2 were tested in Prader-Willi syndrome (PWS) mouse model and allowed to unsilence the expression of normal repressed PWS candidate genes from maternal 15q11-q13 region, whereas in a mouse model of Rubinstein-Taybi syndrome (caused by haploinsufficiency of a histone acetylation writer), the use of HDAC inhibition (HDACi) was found to reverse memory defects (Alarcón et al. 2004; Kim et al. 2020). Second, (2) antisense oligonucleotides (ASOs) may change mRNA and protein expression and are broadly tested for correcting *UBE3A* neuron expression in AS (Bondarev et al. 2021). *UBE3A* neuron expression in AS has been well defined, as it had been shown that the rodent maternal and neuron-specific *Ube3a* expression is mediated by the paternally expressed long-non coding antisense RNA to *Ube3a* (*Ube3a-ATS*) (Meng et al. 2012). ASO enables the *Ube3a-ATS* inhibition or inactivation, resulting in rescuing neurobehavioral and neuropsychological impairments. These can also be achieved by CRISPR/ Cas9 genome editing or CRISPR-mediated RNA editing (Wang and Jiang 2023; Wolter et al. 2020; Schmid et al. 2021). Importantly, various ASO designs are being tested in 4 active phase 1/ 2a clinical trials (NCT05127226, NCT04428281, and NCT04259281). Third, (3) DNA methylation and demethylation modification are applied to treat imprinting disorders caused by duplication of active alleles, such as diabetes mellitus type 1 and Beckwith-Wiedemann syndrome (Carli et al. 2020). Interestingly, another tool, such as CRISPR/ Cas9 mediated epigenome editing, has been developed recently. It enables epigenome manipulation (histone modifications) and targeted gene expression regulation (Nuñez et al. 2021).

## Epigenetics in CPs

The advent of next-generation sequencing (NGS) methods has revolutionized the field of genetics, empowering researchers and clinicians to identify numerous new genes and loci responsible for Mendelian disorders. Recent years have borne witness to remarkable progress in these analyses, driven by advances in omics data technology and bioinformatics, ultimately leading to the recognition of a completely novel category of inborn diseases known as CPs. CPs are defects resulting from germline pathogenic variants within epigenes, leading to aberrant methylation signatures and next to epigenome destabilization (Vos et al. 2023; Sadikovic et al. 2021; Kerkhof et al. 2022).

### Epigenetic modifications in maintaining the genome

The epigenome regulates the chromatin state and, along with the genome, controls the spatial and temporal gene expression in each cell through processes such as DNA methylation, and posttranslational modifications (PTMs) of histones—histone methylation, histone acetylation, histone phosphorylation, and histone ubiquitination, among others (Ludwig and Bintu 2019; Allis and Jenuwein 2016).

The process of DNA methylation is a fundamental and universal epigenetic modification that brings about significant changes in the structural and chemical properties of DNA. This modification has far-reaching impacts on molecular mechanisms such as genomic imprinting, chromatin assembly, and gene transcription (Moylan and Murphy 2016; Petryk et al. 2020). It is essential to development and cellular physiology in humans and other mammals (Petryk et al. 2020). DNA methylation involves the covalent transfer of a methyl group to cytosine followed by guanine (CpG dinucleotides, which cluster in CpG islands). Research has unveiled multiple forms of DNA methylation, including 5-methylcytosine (5mC), 5-hydroxymethylcytosine (5hmC), 5-formylcytosine (5fC), and 5-carboxylcytosine (5caC). Among these, 5mC is the most prevalent epigenetic modification in the human genome and has been extensively studied, while the other forms of DNA methylation are relatively rare and still the subject of ongoing research (Wu et al. 2023; Ma and Kang 2019).

Emil Heitz first reported the structures of condensed heterochromatin (hypermethylated) and open euchromatin (hypomethylated). Heterochromatin is transcriptionally repressed, while euchromatin is permissive to gene transcription (Passarge 1979). The process of DNA methylation is mediated by a group of enzymes known as DNA methyltransferases (DNMTs). DNMTs play a pivotal role in catalyzing the transfer of methyl groups to cytosine residues in DNA. Dysregulation of DNMT activity can lead to abnormal DNA methylation patterns, which are associated with various diseases, including cancer and developmental disorders. Understanding the intricate interplay between DNMTs and the various forms of DNA methylation is a crucial area of research in epigenetics (Edwards et al. 2017; Jin and Robertson 2013).

The pattern of DNA methylation varies throughout the entire genome, with the highest levels occurring in repeated elements, intragenic regions, and genes, while the lowest levels are found in CpG islands. On the molecular level, CpG methylation is integrated within other chromatin modifications, e.g., the histone mark H3K9me3 and CpG methylation frequently co-occur and reinforce one another, while DNA methylation and H3K4me3 exclude one another (Rose and Klose 2014). Importantly, different cell types exhibit distinct methylomes, and methylation patterns change throughout the cellular life, necessitating a robust epigenome maintenance system (Petryk et al. 2020).

Posttranslational modifications (PTMs) of histone proteins are central to the regulation of chromatin structure, playing vital roles in regulating the activation and repression of gene transcription. Numerous PTMs have been identified, including methylation, acetylation, phosphorylation, ubiquitination, sumoylation, biotinylation, ADP-ribosylation, and deamination (Mossink et al. 2021). These PTMs form a so-called histone code that affects the higher chromatin structure, inter-histone interactions, DNA-histone interactions, and the recruitment of non-histone proteins. Moreover, histone writers, erasers, and readers—the protein machinery that adds, removes, or recognizes these PTMs—have become central figures in our understanding of physiological responses in many cell types, including glutamatergic neurons, GABAergic neurons, and glia cells (Nothof et al. 2022).

Histone modifications affect chromatin compaction by two mechanisms. The first mechanism is represented by neutralizing the basic charge of the nucleosome, which loosens inter or intra-nucleosomal DNA-histone interactions. An extensively studied example is the acetylation of lysine residues, which removes the positive charge of lysine and increases the probability of altering the chromatin structural state. The second mechanism relies on non-histone proteins called chromatin-remodeling proteins recruited to the specific PTM (Mossink et al. 2021).

### DNA methylation episignatures in CPs

The DNA methylation analysis is typically conducted on DNA extracted from peripheral blood lymphocytes, commonly utilizing DNA methylation arrays. However, it is worth noting that whole genome bisulfite sequencing can provide accuracy down to a single nucleotide. Researchers revealed the first genome-wide DNA methylation alterations, i.e., episignatures, in 2013 for Intellectual developmental disorder, X-linked syndromic, and Claes-Jensen type (MIM: 314690) resulting from pathogenic variants in *KDM5C* (Grafodatskaya et al. 2013). Since then, episignatures have been described for over 90 conditions, including imprinting defects and a subset of CPs. All known-disease-associated unique episignatures have been cataloged on a free web resource, EpigenCentral, accessed at http://epigen.ccm.sickkids.ca/ (Turinsky et al. 2020).

It has been shown that the DNA methylation patterns are specific for all tested CP cases so far and vary from healthy controls (Kerkhof et al. 2022). Therefore, DNA methylation testing enhances molecular diagnosis and enables the distinction between affected and unaffected individuals as well as between disease-causing and non-disease-causing variants (Vos et al. 2023; Sadikovic et al. 2021; Kerkhof et al. 2022). It also facilitates the functional evaluation of variants of unknown significance and the characterization of newly recognized syndromes, such as Pilarowski-Bjornsonn syndrome and Beck-Fahrner syndrome (Levy et al. 2021; Beck et al. 2020; Pilarowski et al. 2018; Luperchio et al. 2021). Episignature profiling has also been applied in mosaic variants evaluation, including low-level mosaicism, in such epigenes as *KMT2C* and *KMT2D*, resulting in Kleefstra 2 syndrome and Kabuki syndrome 1, respectively (Montano et al. 2022; Oexle et al. 2023). Moreover, DNA methylation biomarkers can also be used for disease monitoring when precision-targeted treatments are applied to reverse the DNA methylation episignature.

On the other hand, Awamleh et al. have pointed to the concept that some DNA methylation patterns may overlap, as shown for epigenes, encoding proteins as part of larger functional complexes. This phenomenon was described for (1) *KDM6A* and *KMT2D* genes encoding different histone modifiers being a part of a multi-protein complex; (2) *EZH2*, *SUZ12*, and *EED*, which are core members of the polycomb repressive complex 2; (3) *ASXL1* and *ASXL2* encoding proteins that function in the polycomb repressive deubiquitinase complex (Choufani et al. 2020; Awamleh et al. 2023; Awamleh et al. 2022). Consequently, the question arose as to whether the current DNA methylation profiling (using methylation arrays) can be applied to distinguish various syndromes or solely to assess specific variants.

## DNA methylation analysis tools

To generate DNA methylation episignatures, patient DNA is isolated from, e.g., peripheral blood. Afterward, it undergoes bisulfite conversion followed by sequencing or methylation profiling using CpG methyl arrays. In the latter up to 850,000 CpG, methylation sites can be analyzed (Nava and Arboleda 2023). Since peripheral blood is easily accessible and cytosine methylation is a highly stable analyte, DNA methylation analysis is adaptable to the routine analytical processes in clinical laboratories. Nevertheless, data analysis is challenging and requires the development of large-scale reference DNA methylation databases, including disorder and tissue-specific reference data sets and controls, and sophisticated analytical processes, including machine learning-based bioinformatic algorithms as analytical classifiers (Sadikovic et al. 2021).

Analytical tools used to establish epigenotypes include (a) machine learning models—a classification model is trained on DNA methylation profiles of the reference or discovery cohorts (cases and controls) at established signature sites. The so-called classifier generates a probability score between 0 and 100% for each variant tested; this score represents the likelihood that a variant is pathogenic and, therefore, matches DNA methylation profiles from individuals with definitive molecular and clinical diagnoses of the neurodevelopmental syndrome in question; (b) principal component analysis (PCA) and hierarchical clustering—the most commonly used model in establishing epigenotypes. Combining these three analytical tools to investigate DNA methylation differences allows a robust interpretation of patient epigenotypes. Once a DNA methylation signature is established, it is annotated to identify the location of sites in the genome, including overlapping genes and specific locations within gene structure such as promoter, transcription start site, gene body, or UTR. Additionally, pathway enrichment and gene ontology can help to elucidate which biological processes and molecular functions genes underlying signature sites are involved in. Finally, comparison to other DNA methylation signatures for CPs can potentially identify common methylation changes and gene targets relevant to exploring therapeutics in the future (Awamleh et al. 2023; Sadikovic et al. 2020). Increasing resolution and specificity ranging from protein complex, gene, sub-gene, protein domain, and even single nucleotide-level Mendelian episignatures have recently been established. In order to develop highly accurate and disease-specific diagnostic classifiers, Levy et al. (2021) proposed the use of (c) multiclass modeling (Levy et al. 2022).

The article of Sadikovic et al. (2021) summarizes the implementation of EpiSign—the genomic DNA methylation testing used to diagnose patients with rare disorders. The implementation and validation of EpiSign was performed across the EpiSign diagnostic laboratory network, comprised of licensed academic nonprofit clinical laboratories in Europe, Canada, and the United States. They used the Illumina Infinium methylation array technology and the EpiSign Knowledge Database (EKD)—a clinical database with >5000 peripheral blood DNA methylation profiles, including disorder-specific reference cohorts and normal controls. Based on comparing each subject individual DNA methylation data and the EKD, the methylation variant pathogenicity (MVP) score was generated, ranging between 0 and 1. A specific EpiSign disorder classification included MVP score assessment with a general threshold of >0.5 for positive, <0.1 negative, and 0.1–0.5 for inconclusive or low confidence. The study provided strong evidence of the clinical utility of EpiSign analysis, with 57 out of 207 clinical samples testing positive for an episignature, giving an overall diagnostic yield of 27.6% (Sadikovic et al. 2021).

Additionally, an ongoing national-scale clinical trial in which episignature analysis in thousands of patients with rare disorders is performed. It is called EpiSign-CAN, and its ultimate goal is to measure the clinical impact of the EpiSign test within Canada. Two groups of patients can be enrolled in this study, namely, first visit patients, i.e., patients who are genetics naïve or have only received standard microarray and/ or fragile X testing, and reflex patients—those who have had extensive genetic testing, including large gene panels and whole exome sequencing, but do not have a diagnosis (https://episign.lhsc.on.ca/can.html).

## Conclusions

In conclusion, the field of CPs has evolved significantly, expanding from its initial association with specific syndromes like Cornelia de Lange syndrome to encompass a diverse range of genetic disorders resulting from chromatin state imbalances. With the advent in CPs recognition, we have also witnessed the development of episignature profiling methods that has become a tool for the evaluation of variants of unknown significance and the differentiation of specific CP types. Moreover, it has been shown that episignatures hold great potential for pioneering targeted therapeutic interventions. It is noteworthy that the applicability of epigenome-targeting approaches extends beyond CPs and encompasses a wide spectrum of both cancer and non-cancer diseases. There have been reports of successful drug regimens targeting epigenome components or harnessing the power of epigenome editing in diverse disease contexts. Given the rapid advancements in this field, it is highly probable that targeted therapies for CPs will be developed, and next, implemented to the clinics.

### Supplementary materials

ESM 1 Supplementary Table 1 The list of ongoing or finished clinical trials regarding imprinting defects or chromatinopathies (XLSX 44 kb)

## Data Availability

https://clinicaltrials.gov/ http://epigen.ccm.sickkids.ca https://www.omim.org/
